# *Staphylococcus aureus* Population Structure and Genomic Profiles in Asymptomatic Carriers in Switzerland

**DOI:** 10.3389/fmicb.2020.01289

**Published:** 2020-06-24

**Authors:** Danai Etter, Sabrina Corti, Simona Spirig, Nicole Cernela, Roger Stephan, Sophia Johler

**Affiliations:** ^1^Institute for Food Safety and Hygiene, Vetsuisse Faculty, University of Zurich, Zurich, Switzerland; ^2^Institute of Food, Nutrition and Health, Department of Health Science and Technology, ETH Zürich, Zurich, Switzerland

**Keywords:** nasal colonization, MRSA, *pvl*, farmers, livestock, community, hospital, companion animals

## Abstract

*Staphylococcus aureus* is a leading cause for clinical infections and food intoxications, causing over 100,000 yearly cases of bacteremia in the United States and 434 food-borne outbreaks in the European Union. Approximately 30% of the population permanently carry *S. aureus* asymptomatically in their nasal cavity. The risk of infection and transmission to food items or the environment is higher in individuals that are nasally colonized. In addition, *S. aureus* can acquire various antimicrobial resistances leading to therapeutic failure, additional medical costs, and fatalities. Methicillin-resistant *S. aureus* (MRSA) cause a considerable burden of disease in humans and animals. MRSA carriage has been associated with animal and in particular livestock contact. Extensive current data on the virulence gene profiles, as well as data on antimicrobial resistance determinants is crucial in developing effective strategies to mitigate the burden of disease. To this end, we screened the anterior nares of 160 test subjects (87 pupils and 73 members of farmer families) in Switzerland for *S. aureus* carriage. A total of 73 *S. aureus* isolates were obtained. Factors such as exposure to farm or companion animals and personal medical history were recorded using a questionnaire. Using a DNA microarray, isolates were assigned to clonal complexes (CCs), and virulence and resistance gene profiles were determined. The collected strains were assigned to 20 CCs, among others CC1, CC7, CC8, CC15, CC30, CC45, CC97, and CC398. Two MRSA strains and one multiresistant isolate carrying genes *blaZ/I/R*, *InuA*, *aadD*, *tetK*, and *fosB* were isolated from farmers with intensive exposure to animals. Strains carrying *pvl*, causing severe skin lesions and necrotizing pneumonia, as well as tetracycline, erythromycin, and kanamycin resistance genes were found in individuals that had taken antibiotics during the last year. A variety of superantigenic toxin genes was detected, including among others, the toxic shock syndrome toxin (*tst1*), and various enterotoxins (*sea*, *sec*, *sel*, and the *egc* cluster). Contact to chickens was identified as a significant factor contributing to *S. aureus* colonization.

## Introduction

*Staphylococcus aureus* is a human pathogen, resulting in infections and foodborne intoxications. It caused over 100,000 cases of bacteremia in the United States in 2017 and 434 food-borne outbreaks in the European Union in 2015 (Tong et al., 2015; EFSA, 2016; Kourtis et al., 2019). Furthermore, it can colonize human anterior nares as a commensal bacterium. Around 20–30% of the population permanently carry *S. aureus* asymptomatically in their nose (Sakr et al., 2018), another 20–60% can be intermittent carriers (Kluytmans et al., 1997; Wertheim et al., 2005). Initial colonization may take place during the first days of life via transmission from mother to child (Maayan-Metzger et al., 2017). After birth the hands have been identified as the main source of transmission for the pathogen to colonize the nose (Wertheim et al., 2005). The nasal habitat may act as a reservoir for pathogen spread and can pose a threat to the carrier itself (Nouwen et al., 2006). An elevated risk of infection associated with persistent nasal carriage of *S. aureus* in surgical or dialysis patients emphasizes the importance of characterizing nasal colonization and associated risk factors (Kluytmans et al., 1997; Nouwen et al., 2006). In addition, antimicrobial resistance among *S. aureus* is rapidly emerging globally and rendering treatment of chronic and acute *S. aureus* infections increasingly difficult. The alarming prospect of a post-antibiotic era highlights the importance of identifying new therapeutic agents (Johler, 2017; Vestergaard et al., 2019). Specific virulence factors and/or master virulence regulators have been suggested as promising targets (Maura et al., 2016; Ansari et al., 2019). In particular, methicillin-resistant *S. aureus* (MRSA) have been studied in recent years with regard to their transmission dynamics (Christopher et al., 2011; Davis et al., 2012). Forms of transmission include hospital-acquired, community-acquired, and livestock-acquired with the MRSA in question being designated as HA-MRSA, CA-MRSA, and LA-MRSA, respectively (Deurenberg and Stobberingh, 2008; Knox et al., 2015; Smith, 2015; Kwok et al., 2018). Intensive contact to animals has been shown to increase the risk of LA-MRSA carriage among farmers, veterinarians, and animal owners (Graveland et al., 2011; Davis et al., 2012; Fluit, 2012). Several studies have investigated *S. aureus* colonization of veterinarians (Huber et al., 2011; Rosenkranz Wettstein et al., 2014), on-farm and food-chain epidemiology (Kraemer et al., 2017; Leuenberger et al., 2019; Morach et al., 2019), or colonization in the general population in Switzerland (Mégevand et al., 2010; Olearo et al., 2016). Here, we aim to provide insight into colonization patterns in asymptomatic carriers with varying animal exposure. We screened 160 individuals for nasal *S. aureus* and collected data on animal contact and medical history using a questionnaire. The obtained isolates were characterized by DNA microarray, which allows for assignment of clonal complexes (CCs), and determines the presence of resistance and virulence genes.

## Materials and Methods

### Questionnaire

A questionnaire was used to collect metadata. We assessed exposure of participants to farm and companion animals, medical history including antibiotic prescriptions and hospital or doctor visits. Answers concerning exposure to animals ranged from never to daily (0, 1, 2, 3, 4, 5). Medical history was recorded to assess, whether the individual had been to a doctor/hospital and/or received antibiotics in the last year (0/1). Raw data can be examined in Supplementary Table S2.

### Sampling, Bacterial Isolation, Species Identification, and DNA Extraction

Each volunteer (87 pupils and 73 members of farmer families) provided a written declaration of consent and took his/her own nasal swab after prior demonstration of the sampling procedure. All nasal swabs were streaked on EASY Staph^®^ plates (BIOKAR Diagnostics, Beauvais, France) and incubated at 37°C for 48 h. Presumptive *S. aureus* colonies were confirmed using matrix assisted laser desorption/ionization—time of flight mass spectrometry (MALDI-TOF MS, Bruker, Fällanden, Switzerland). After cell lysis with Lysostaphin (Sigma–Aldrich, Buchs, Schweiz), DNA was extracted with the Blood & Tissue Kit (Qiagen, Hombrechtikon, Switzerland).

### DNA Microarray Analysis

DNA microarray was performed using Staphytype genotyping kit 2.0 (Alere, Wädenswil, Switzerland) following the manufacturer’s instructions. An ArrayMate reader (Alere) was used for signal acquisition. In addition, the similarity of the virulence and resistance gene profiles was visualized using SplitsTree4^1^ (Huson and Bryant, 2006) as previously described (Wattinger et al., 2012). Readouts can be found in Supplementary Table S1.

### Statistical Analysis

Binomial and multinomial logistic regression analyses were performed to ascertain the effects of age, gender, profession, medical history, and exposure to farm and companion animals on the likelihood that participants are colonized with *S. aureus*, with certain CCs or harbor MRSA strains or enterotoxin gene carrying isolates (SPSS, v26.0.0.0). *P*-values < 0.05 were considered significant (Supplementary Table S4).

## Results

### *S. aureus* Prevalence and Clonal Complexes in Sampled Populations

**FIGURE 1:**
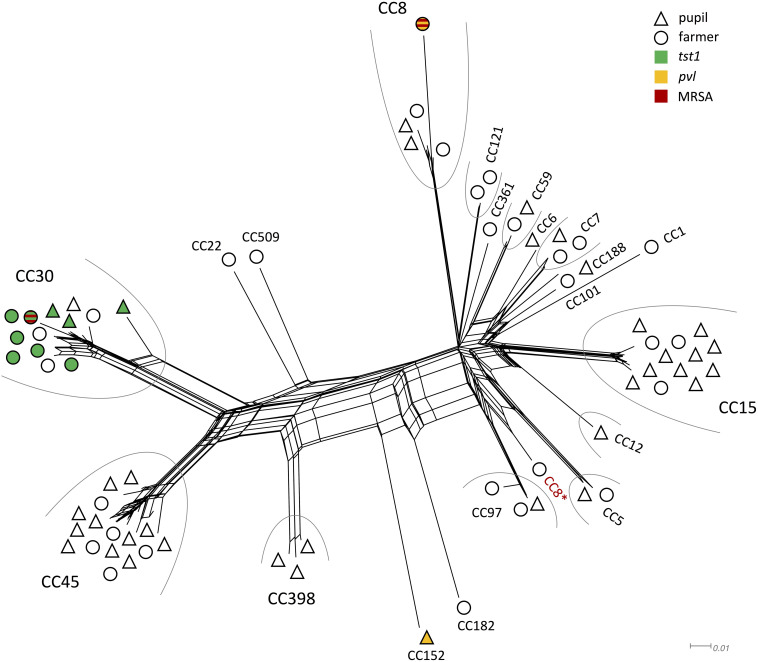
Splitstree showing the similarity of genomic profiles determined by microarray. Clonal complexes are grouped by an arc, if there were at least two isolates of the same CC. Symbol shape denominates whether the isolate originated from a farmer (O) or pupil (Δ). The fill color indicates presence of *tst1* (green), *pvl* (yellow), or MRSA (red). ^∗^CC8 strain that grouped differently than other CC8 strains.

Of the 160 nasal swabs from asymptomatic carriers 46% (*n* = 73, confidence interval = 7.72) were positive for *S. aureus*. In the sampled 87 pupils and 73 family members of farmers colonization rates slightly differed with 44% in pupils and 48% in family members of farmers. The most common CCs for pupils were CC45 (*n* = 11) and CC15 (*n* = 10), while for family members of farmers, it were mainly CC30 (*n* = 9) and CC45 (*n* = 5) (Table 1). Some CC were exclusively found in one of the two groups, e.g., CC1, CC22, CC101, CC121, CC182, CC361, and CC509 in family members of farmers, while CC6, CC12, CC152, CC188, and CC398 were only found in pupils (Figure 1). All other strains belonged to CC5, CC7, CC8, CC59, and CC97.

**TABLE 1:** Clonal complexes found in nasal samples from farmers and pupils.

Clonal complex	Farmers	Pupils	Total	Resistance genes^a^	Enterotoxin genes^a^	Comments^a^
CC1	1		1	*fusC*, *blaZ/I/R*	*sea*^b^, *seb*, *seh*, *sek, seq*	
CC5	1	1	2	*blaZ/I/R*, *fosB*	*sed*, *sej*, *ser*, *egc*^c^	
CC6		1	1	*fosB*	*sea*^b^	
CC7	2	1	3	*blaZ/I/R* (2)	*sea*^b^	
CC8	4	2	6	*mecA* (1), *blaZ/I/R* (3), *ACME* (1), *msr(A)* (1), *mph(C)* (1), *aphA3* (1), *fosB*	*sea*^b^ (2), *sed* (1), *sej* (1), *sek* (2), *seq* (2), *ser* (1), *egc*^c^ (1)	MRSA (*mecA*) and *pvl* (1)
CC12		1	1	*blaZ/I/R*, *fosB*	*sea*^b^	
CC15	3	10	13	*blaZ*/I/R (12), *InuA* (1), *aadD* (1), *tetK* (1), *fosB*		
CC22	1		1	*blaZ/I/R*	*egc*	
CC30	9	4	13	*mecA* (1), *blaZ/I/R*, *ermA* (2), *fosB*	*tst1* (9), *sea*^b^ (5), *sec* (1), *sel* (1), egc	MRSA (*mecA*) (1)
CC45	5	11	16	*blaZ/I/R* (8)	*sec* (10), *seg* (1), *sel* (10), *egc*^c^	
CC59	1	1	2	*ermA* (1)		
CC97	2	1	3			
CC101	1		1	*blaZ/I/R*, *fosB*		
CC121	2		2	*blaZ/I/R*, *fosB*	*egc*^c^	
CC152		1	1	*blaZ/I/R*, *tetK*, *qacA*		*pvl*
CC182	1		1		*seh*, *egc*^c^	
CC188		1	1	*blaZ/I/R*		
CC361	1		1	*blaZ/I/R*, *fosB*	*egc*^c^	
CC398		3	3	*blaZ/I/R* (2), *qacC* (1)		
CC509	1		1	*blaZ/I/R*	*egc*^c^	
Total	35	38	73			

*Resistance and enterotoxin genes found by microarray are listed, grouped by CC. ^a^If not all isolates assigned to the respective CC harbored a gene, the number of positive isolates is indicated in brackets. ^b^Either sea or allelic variant seaN315 was found, the respective variant is indicated in Supplementary Table S3. ^c^Enterotoxin gene cluster (egc) containing genes: seg, sei, sem, sen, seo, seu.*

### Exposure to Animals and Medical History of Participants

Overall animal exposure varied greatly between the two populations. Family members of farmers had an average exposure value of 2.4, corresponding to monthly-to-weekly exposure. Pupils on the other hand only reached a value of 0.8, corresponding to never-to-yearly exposure. Almost all family members of farmers were in contact with cattle on a daily basis (∅ = 4.8). Binomial logistic regression analysis revealed that exposure to chickens correlated significantly with whether an individual was colonized with *S. aureus* or not (*p* = *0.019*). No association between exposure to certain animals and the occurrence of specific CC could be found. Medical history including visits to doctors or hospitals, as well as antibiotic prescription was not significantly associated with neither *S. aureus* carriage, nor occurrence of resistance in this study.

### Similarity of Genomic Profiles

All isolates grouped according to their respective CC, with the exception of one CC8 strain that clustered closely with CC5 and CC97 strains (Figure 1). The two MRSA strains grouped very far from each other. The same applies for the two *pvl* positive strains. Genomic profiles of CC1, CC6, CC7, CC59, CC101, CC121, CC188, and CC361 all clustered closely.

### Resistance Genes

A broad variety of resistance genes was found among isolates. The most common resistance genes were *blaZ/I/R* (*n* = 52) conferring *β*-lactam resistance and *fosB* (*n* = 40) coding for a metallothiol transferase, followed by *ermA* (*n* = 3) an erythromycin resistance gene, and *tetK* (*n* = 2) coding for tetracycline resistance. Two strains were identified as MRSA (CC8, CC30), both harboring the *mecA* cassette. Both were isolated from family members of farmers (Figure 1).

Other resistance genes included the arginine catabolic mobile element (ACME) that provides multiple immune modulating functions including resistance to polyamines (Joshi et al., 2012). A fusidic acid resistance protein *fusC*, and the genes *msr(A)* and *mph(C)* both coding for macrolide efflux pumps (Ross et al., 1995; Matsuoka et al., 2003) were observed. Genes *aadD* and *aphA3* conferring resistance to aminoglycosides such as kanamycin and gentamycin were found. Additionally, *InuA* a gene responsible for lincosamide modification, and *qacA* and *caqC* encoding antiseptic resistance were discovered (Supplementary Table S3).

### Enterotoxin Genes and Other Virulence Factors

Of the 73 isolates 48 isolates (66%) carried at least one enterotoxin gene (Table 1). Two isolates (CC8, CC152) carried the virulence factor panton-valentine leukocidin (*pvl*) gene that is responsible for severe skin lesions and necrotizing pneumonia (Lina et al., 1999; Kaneko and Kamio, 2004). One of the obtained *pvl* isolates was identified as an MRSA strain (Figure 1). All strains harboring the *tst1* gene (*n* = 9) were assigned to CC30 (Figure 1). The most common enterotoxin genes were the genes grouped in the enterotoxin gene cluster *egc*: *seg*, *sei*, *sem*, *sen*, *seo*, *seu* (*n* = 38), *sea* (*n* = 13), *sec* (*n* = 11), and *sel* (*n* = 11). Other enterotoxin genes included *seb* (*n* = 1), *sed* (*n* = 3), *seh* (*n* = 2), *sej* (*n* = 3), *sek* (*n* = 3), and *seq* (*n* = 3).

## Discussion

The observed colonization frequency of 46% (44% in pupils and 48% in family members of farmers) is in accordance with current estimates for nasal carriage rates (Sakr et al., 2018). When we compared the genomic profiles of all isolates, there were no overlaps, meaning that all isolates were unique in their genomic profile and therefore not clonal. All big clusters of CCs with more than three isolates were found in both family members of farmers and pupils. The large clusters of CC8, CC15, CC30, and CC45 comprised 66% of isolates and all represent community or hospital associated complexes (Pérez-Montarelo et al., 2017). One CC8 strain differed from all other detected CC8 strains in view of its enterotoxin gene profile. While other CC8 strains harbored diverse enterotoxins, the outlier strain only carried *egc*. Strains carrying *egc* were shown to be more common in asymptomatic carriers than in infections (Van Belkum et al., 2006). Similar distributions have been reported (Van Belkum et al., 2006; Mohamed et al., 2019). Surprisingly, strains of CC398 were exclusively found in pupils with very limited farm animal contact (once a year or less), although this CC has regularly been linked to livestock (Verkade and Kluytmans, 2014). This may be a first indicator for a possible shift from livestock-acquired to community-acquired transmission. Most other complexes were associated with isolates from both populations. This is consistent with findings of previous studies for various CCs including CC5, CC7, CC59, and CC97 (Nulens et al., 2008; Coombs et al., 2010; Budd et al., 2015; Wang et al., 2017; Challagundla et al., 2018).

Chickens were identified as a significant factor for whether an individual was colonized with *S. aureus* or not. No significant factors that correlated with colonization of certain CC, resistance genes, or toxin profiles could be determined. This was likely due to limitations in sample size and overall low number of strains exhibiting resistance/toxin genes. It is, however, noteworthy that one of the two MRSA strains was isolated from an individual that had taken antibiotics during the last year. The same was the case for both strains harboring *pvl*, both strains harboring *qacA/C*, and one of the strains carrying *ermA*. Increased occurrence of these genes in HA-strains has been reported (Lina et al., 1999; Schmitz et al., 2000; Mayer et al., 2001). An unusual accumulation of CC152 *pvl* strains has been reported in African refugees in Switzerland, possibly representing an emerging genotype (Jaton et al., 2016). One of two MRSA strains (CC8) that was isolated from a family member on a farm with intensive contact to cattle, pigs, and chicken possessed additional genes coding for *pvl*, ACME, *blaZ/I/R*, *msr(A)*, *mph(C)*, *aphA3*, and *fosB*. This represents a combination very similar to USA300 MRSA, a CA-MRSA strain often encountered in the US and considered a “superbug” (Monecke et al., 2011; Smith, 2015; Planet, 2017). Similar strains were found in Swiss hospital patients accounting for approximately 10% of the encountered MRSA (Seidl et al., 2014). The second MRSA strain featured genetic elements that are typical for CA- or HA-acquired MRSA of CC30, including the presence of *blaZ/I/R*, *ermA*, *fosB*, *egc*, and *tst1* (Monecke et al., 2011). Another strain belonging to CC15 was classified as multiresistant according to international standard definitions for acquired resistance (Magiorakos et al., 2012) by carrying *blaZ/I/R*, *InuA*, *aadD*, *tetK*, and *fosB*. All three multiresistant strains were isolated from individuals regularly exposed to animals. While the DNA microarray data presented in this study provide new insights into the genetic structure of the tested isolates, it should not be used to extrapolate phenotypic traits.

This study provides an overview of *S. aureus* isolates found in asymptomatic carriers with varying exposure to animals. A diverse set of strains was characterized in terms of population structure and presence of virulence and resistance determinants. Our findings suggest that exposure to chicken may increase the risk of *S. aureus* nasal colonization and that intensive animal exposure may represent a risk factor for acquisition of multiresistant strains.

## Data Availability Statement

The original contributions presented in the study are publicly available. These data can be found here: https://www.ebi.ac.uk/arrayexpress/. Accession number: E-MTAB-8960.

## Ethics Statement

Ethical review and approval was not required for the study on human participants in accordance with the local legislation and institutional requirements. The patients/participants provided their written informed consent to participate in this study.

## Author Contributions

SC and RS contributed to conception and design of the study. DE analyzed the data and wrote the first draft of the manuscript. SS, SC, and NC were responsible for data acquisition. DE, SJ, and RS wrote sections of the manuscript. All authors contributed to manuscript revision and read and approved the submitted version.

## Conflict of Interest

The authors declare that the research was conducted in the absence of any commercial or financial relationships that could be construed as a potential conflict of interest.

## Footnotes

1 http://www.splitstree.org/

## Supplementary Material

TABLE S1 Microarray profile raw data. The outcome of every hybridization reaction is shown as either positive, negative, or ambiguous as determined by the ArrayMate reader (Alere).

TABLE S2 Questionnaire raw data. Answers to the questionnaire are provided as raw data.

TABLE S3 Strains. List of all isolates with corresponding CCs, resistance genes, and toxin profile.

TABLE S4 Statistics. Binomial regression analysis output from SPSS.
